# Genotypes and haplotypes of the *VEGF *gene and survival in locally advanced non-small cell lung cancer patients treated with chemoradiotherapy

**DOI:** 10.1186/1471-2407-10-431

**Published:** 2010-08-16

**Authors:** Xiaoxiang Guan, Ming Yin, Qingyi Wei, Hui Zhao, Zhensheng Liu, Li-E Wang, Xianglin Yuan, Michael S O'Reilly, Ritsuko Komaki, Zhongxing Liao

**Affiliations:** 1 Department of Epidemiology, The University of Texas M. D. Anderson Cancer Center, 1515 Holcombe Blvd, Houston, Texas 77030, USA; 2 Department of Radiation Oncology, The University of Texas M. D. Anderson Cancer Center, 1515 Holcombe Blvd, Houston, Texas 77030, USA

## Abstract

**Background:**

Vascular endothelial growth factor (VEGF) is a major mediator of angiogenesis involving in carcinogenesis, including lung cancer. We hypothesized that *VEGF *polymorphisms may affect survival outcomes among locally advanced non-small cell lung cancer (LA-NSCLC) patients.

**Methods:**

We genotyped three potentially functional *VEGF *variants [-460 T > C (rs833061), -634 G > C (rs2010963), and +936 C > T (rs3025039)] and estimated haplotypes in 124 Caucasian patients with LA-NSCLC treated with definitive radiotherapy. We used Kaplan-Meier log-rank tests, and Cox proportional hazard models to evaluate the association between *VEGF *variants and overall survival (OS).

**Results:**

Gender, Karnofsky's performance scores (KPS) and clinical stage seemed to influence the OS. The variant C genotypes were independently associated with significantly improved OS (CT+CC vs. TT: adjusted hazard ratio [HR] = 0.58; 95% confidence interval [CI] = 0.37-0.92, *P *= 0.022), compared with the *VEGF *-460 TT genotype.

**Conclusions:**

Our study suggests that *VEGF *-460 C genotypes may be associated with a better survival of LA-NSCLC patients after chemoradiotherapy. Large studies are needed to confirm our findings.

## Background

Non-small cell lung cancer (NSCLC) accounts for 89% of all lung cancer, and 30% of NSCLC patients present with locally-advanced unresectable tumors (unresectable stage IIIa and IIIb) [1]. Radiotherapy combined with chemotherapy, either sequentially or concurrently, is the standard treatment regimen for these patients, which, however, have resulted in unsatisfactory prognosis, with a 5-year survival rate of about 10-15% [1], and a median survival time (MST) of 16-18 months [2,3]. There has been a persistent interest in search for readily accessible molecular markers that may provide therapeutic benefits by predicting clinical outcomes of these locally advanced NSCLC (LA-NSCLC) on an individual basis.

Angiogenesis is an essential process in the development, growth, and metastasis of malignant tumors including lung cancer [4]. Vascular endothelial growth factor (VEGF) is one of the most potent and predominant mediators of angiogenesis, which stimulate vascular endothelial cell growth, survival, and proliferation. Recent investigation has further revealed that VEGF acts as a mitogenic and survival signal for the tumor cell itself, indicating a broader range of tumor-promoting effects. Therefore, VEGF stands as a good candidate for prognostic biomarkers in cancer patients. Indeed, most tumors produce VEGF, whereas inhibition of the VEGF signaling significantly inhibits tumor growth *in vivo *[5]. In NSCLC, it has been found that a high expression of VEGF protein or mRNA was associated with increased tumor angiogenesis, early tumor relapse and reduced survival time [6-8]. A recent report further linked some functional *VEGF *polymorphisms with prognosis of early stage (stage I and II) NSCLC, probably through regulation of VEGF expression [9]. However, LA-NSCLC accounts for a significant proportion of lung cancer, and it is not known if *VEGF *polymorphisms are associated with prognosis within this particular population.

Previous studies primarily focused on three common functional single nucleotide polymorphisms of the *VEGF *gene, including the -460 T > C, -634 G > C (also assigned as +405 G > C) and +936 C > T (minor allele frequency = 0.422, 0.431 and 0.222 in Caucasians, respectively, according to the Hapmap database). The -460 T > C SNP is located in the promoter region and may influence the promoter activity [10]; the -634 G > C SNP lies within the 5'-untranslated region and may affect the transcriptional factor binding affinity [11]; the +936 C > T SNP is located in the 3'-untranslated region and has been associated with lower VEGF plasma levels [12]. In the present study, we evaluated the association of these three potentially functional *VEGF *SNPs (i.e., -460 T > C, -634 G > C [also assigned as +405 G > C] and +936 C > T) with overall survival (OS) of LA-NSCLC patients.

## Methods

### Study populations

Clinical data were derived from a large dataset of 576 NSCLC patients established at The University of Texas M. D. Anderson Cancer Center (Houston, TX), in which patients were recruited and histologically confirmed between Oct. 1998 and Nov. 2006. Details of this study population have been described previously [13]. Briefly, this analysis consisted of 124 Caucasian patients with stage IIIa or IIIb NSCLC according to the TNM staging system, a relatively homogenous group that was treated by chemoradiotherapy. Those patients who had surgical resection, or had been treated elsewhere before coming to M. D. Anderson were excluded from the analysis. The study protocol was approved by the M. D. Anderson Cancer Center institutional review board and informed consents were waived. We complied with Health Insurance Portability and Accountability Act (HIPAA) regulations.

### Genotyping

Genomic DNA was extracted from the buffy coat fraction of each blood sample by using a Blood Mini Kit (Qiagen, Valencia, CA) according to the manufacturer's instructions. DNA purity and concentrations were determined by spectrophotometric measurement of absorbance at 260 and 280 nm by UV spectrophotometer. The selected three *VEGF *SNPs (-460 T > C/rs833061, -634 G > C/rs2010963, and +936 C > T/rs3025039) were genotyped using the polymerase chain reaction (PCR) -restriction fragment length polymorphism (RFLP) method. The PCR primers used for -460 T > C, -634 G > C, and +936 C > T polymorphisms were 5'-CTCTTTAGCCAGAGCCGGGG-3' (forward) and 5'-TGGCCTTCTCCCCGCTCCGAC-3' (reverse); 5'-CGACGGCTTGGGGAGATTGC-3' (forward) and 5'-GGGCGGTGTCTGTCTGTCTG-3' (reverse); and 5'-AGGGTTCGGGAACCAGATC-3' (forward) and 5'-CTCGGTGATTTAGCAGCAAG-3' (reverse), respectively. The following PCR conditions were performed: one cycle at 95°C for 5 min; 35 cycles of 95°C for 30 s, 60°C for 30 s, and 72°C for 45 s; and a final extension at 72°C for 10 min. The PCR products were studied after digestion with *Bsa*HI, *Bsm*FI, and *Nla*III restriction enzymes. Genotypes of these *VEGF *SNPs were determined as previously reported [14]. For the PCR-RFLP-based genotyping assay, two research assistants independently read the gel pictures, and the genotyping was repeated if there was a disagreement of the result. We selected 10% of the samples for replication, and the results were 100% concordant.

### Statistical analysis

The two-sided χ^2 ^and Student *t *tests were performed to determine any statistically significant differences in the distributions of the *VEGF *genotypes by the demographic variables and clinical features. We used the Kaplan-Meier estimates to evaluate OS among three genotype groups, and the log-rank test to test for equality of the survival distributions. We conducted univariate analysis and used multivariate Cox proportional hazard models to estimate the effect of each SNP on survival with or without other confounding factors. Haplotype frequencies and individual haplotypes were generated using SAS PROC HAPLOTYPE. The associations between haplotype and overall survival (OS) were determined using a dominant genetic model to preserve statistical power. All analyses were performed using SAS software (version 9.1; SAS Institute, Cary, NC).

## Results

### Population characteristics

Clinical and pathological characteristics of the patients included in current study are shown in Table 1. Among the 124 patients, there were 67 males (54%) and 57 females (46%), whose ages ranged from 35 to 88 years. There were 40 (32.3%) adenocarcinoma, 38 (31.6%) squamous cell carcinoma and 46 (37.1%) large cell carcinoma. All patients received radiotherapy, delivered as 1.8 to 2 Gy per fraction once a day with a total median radiation dose of 66 Gy (ranging between 50 and 72 Gy). A number of 112 (90.3%) patients also received platinum plus taxane or etoposide-based chemotherapy. At the end of follow-up, 92 (74.2%) patients died, and the MST was 17 months (ranging between 1 and 97 months) in the overall study subjects.

**Table 1:** Characteristics of the study population (n = 124) and overall survival

Characteristics	No. of Patients (%)	No. of Deaths (%)*	*P*^†^	MST (95% CI, month)	*P*^‡^
**Age**			0.456		0.468
≤ 60 years	55 (44.4)	39 (70.9)		21.0 (13.0-32.0)	
> 60 years	69 (56.6)	53 (76.8)		18.0 (13.0-26.0)	
**Gender**			0.175		**0.046**
Female	57 (46.0)	39 (68.4)		26.0 (17.0-38.0)	
Male	67 (54.0)	53 (79.1)		17.0 (12.0-22.0)	
**Smoke**			0.752		0.679
Ever	114 (91.9)	85 (74.6)		20.0 (16.0-26.0)	
Never	10 (8.1)	7 (70.0)		16.0 (11.0-18.0)	
**Pack -year**			0.542		0.861
≤ 40	60 (48.4)	46 (75.0)		19.0 (14.0-29.0)	
> 40	64 (51.6)	46 (73.0)		20.0 (13.0-29.0)	
**Histology**			0.828		0.632
Adenocarcinoma	40 (32.3)	31 (77.5)		20.0 (17.0-31.0)	
Squamous cell	38 (31.6)	28 (73.7)		16.0 (12.0-25.0)	
Others	46 (37.1)	33 (71.7)		24.0 (13.0-36.0)	
**KPS**			0.175		0.067
90-100	38 (30.7)	24 (66.2)		22.0 (16.0-39.0)	
80	67 (54.0)	53 (79.1)		20.0 (14.0-27.0)	
< 80	19 (15.3)	15 (79.0)		13.0 (8.0-29.0)	
**Stage**			0.057		0.088
IIIa	52 (41.9)	34 (65.4)		20.0 (16.0-39.0)	
IIIb	72 (58.1)	58 (80.6)		17.0 (13.0-23.0)	
**Chemotherapy**			0.186		0.766
Yes	112 (90.3)	85 (75.9)		20.0 (16.0-26.0)	
No	12 (9.7)	7 (58.3)		17.0 (11.0-44.0)	
**Radiotherapy dose**			0.360		0.857
< 70Gy	59 (47.6)	46 (78.0)		21.0 (16.0-33.0)	
≥ 70Gy	65 (52.4)	46 (70.8)		17.0 (12.0-24.0)	

Abbreviations: MST, median survival time.

* Percentage of deaths in each stratum.

^† ^Chi -square test for difference in the distribution of deaths.

^‡ ^Log-rank test for survival time in the univariate analysis.

**Figure 1:**
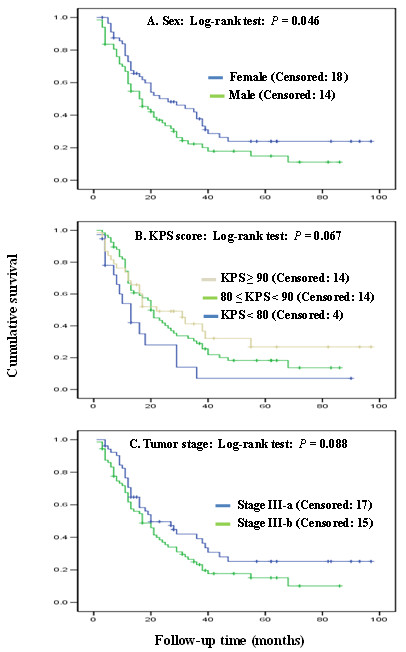
**Overall survival curves by selected host factors with an association of significance level**. The P values were obtained from the unadjusted Log-rank test.

To determine if there was any confounding factor influencing patients' death or survival time, we performed the chi-square test and univariate analysis of Log-rank test for the relationship of death number and OS with clinicopathologic characteristics. We did not find any significant difference in the death distribution by different clinicopathologic characteristics. However, gender seemed to be a confounding factor of OS (MST: 17 months of male vs. 26 months of female, *P *= 0.046) (Figure 1A), whereas age, smoking status, pack-year, histology, application of chemotherapy, and radiation dose did not. Notably, Karnofsky's performance scores (KPS) and clinical stage showed a marginally significant association with OS (*P *= 0.067 and *P *= 0.088) (Figure 1B and 1C), suggesting they might be additional confounding factors required for control.

### *VEGF* genotypes and NSCLC survival

**Figure 2:**
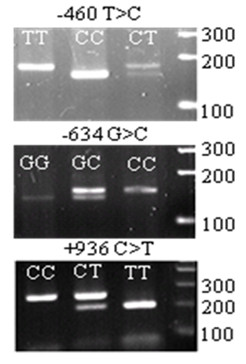
**PCR-based restriction analysis of the *VEGF* SNPs shown on agarose electrophoresis**.

**Figure 3:**
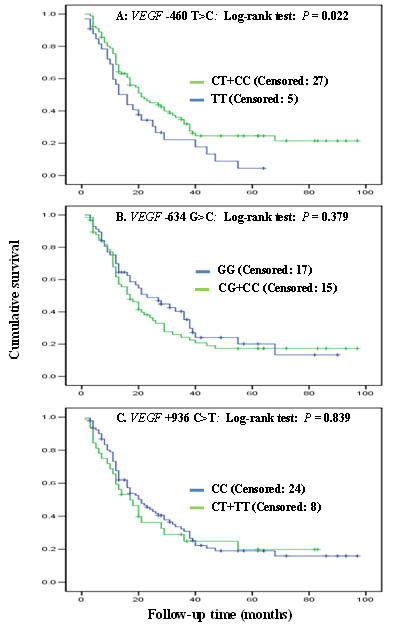
**Overall survival curves by genotypes of *VEGF* gene**. The P values were obtained from the Cox model with adjustment for age, sex, smoking status, tumor histology, KPS score, tumor stage, use of chemotherapy and radiotherapy dose.

The representative PCR-based restriction analyses for the *VEGF *-460 T > C, -634 G > C, and +936 C > T polymorphisms were shown in Figure 2. The genotype distributions of the three *VEGF *SNPs and the association with OS are summarized in Table 2. In all patient, the -460 CT genotype (n = 67) was associated with a significantly increased OS (crude hazard ratio [HR] = 0.60; 95% confidence interval [CI], 0.37-0.95, *P *= 0.031), compared with the *VEGF *-460 TT genotype (n = 33). After a multivariate adjustment with age, sex, smoking status, tumor histology, KPS score, tumor stage, history of chemotherapy, and radiation dose, the HR remained statistically significant (adjusted HR = 0.56; 95% CI, 0.34-0.90, *P *= 0.018; Table 2). Although the homozygous CC genotype showed a tendency favoring increased OS, there was no statistical significance, probably because of a reduced detecting power resulting from small sample size (n = 24). Therefore, we combined the CT and CC genotypes for additional analysis. Under this dominant genetic model, the combined -460 CT/CC variant genotypes (n = 91) were associated significantly with improved OS (adjusted HR = 0.58; 95% CI = 0.37-0.92, *P *= 0.022) (Figure 3A), compared with the *VEGF *-460 TT genotype (n = 33). For the other two *VEGF *SNPs -634 G > C and +936 C > T, we repeated the analyses but did not find any significant associations under either the additive model or dominant model (Table 2 and Figure 3B and 3C).

**Table 2:** Association between *VEGF *genotypes and overall survival

Genotypes	Case No.	Deaths No. (%)*	MST (95% CI, month)	Crude HR (95% CI)	*P*^†^	Adjusted HR (95% CI)	*P*^‡^
***VEGF *-460T > C (rs833061)**							
TT	33	28 (84.9)	16.0 (11.0-25.0)	1.00		1.00	
CT	67	47 (70.2)	21.0 (17.0-32.0)	**0.60 (0.37-0.95)**	**0.031**	**0.56 (0.34-0.90)**	**0.018**
CC	24	17 (70.8)	27.0 (10.0-36.0)	0.75 (0.41-1.37)	0.347	0.67 (0.36-1.26)	0.212
CT+CC	91	64 (70.3)	21.0 (17.0-31.0)	**0.63 (0.40-0.99)**	**0.043**	**0.58 (0.37-0.92)**	**0.022**
***VEGF* -634G > C (rs2010963)**							
GG	57	40 (70.2)	23.0 (16.0-36.0)	1.00		1.00	
CG	41	34 (82.9)	17.0 (13.0-25.0)	1.20 (0.76-1.90)	0.440	1.17 (0.74-1.88)	0.502
CC	26	18 (69.2)	12.0 (9.0-29.0)	1.25 (0.71-2.18)	0.436	1.28 (0.72-2.28)	0.399
CG+CC	67	52 (76.1)	17.0 (12.0-24.0)	1.22 (0.80-1.84)	0.356	1.21 (0.79-1.84)	0.379
***VEGF* +936C > T (rs3025039)**							
CC	92	68 (73.9)	21.0 (16.0-29.0)	1.00		1.00	
CT	30	22 (73.3)	17.0 (11.0-29.0)	1.08 (0.67-1.75)	0.742	1.00 (0.60-1.66)	0.992
TT	2	2 (100)	14.5 (9.0-20.0)	2.14 (0.52-8.80)	0.291	1.95 (0.46-8.29)	0.367
CT+TT	32	24 (75.0)	17.0 (11.0-28.0)	1.13 (0.71-1.80)	0.606	1.05 (0.65-1.71)	0.839

Abbreviation: MST, median survival time.

* Percentage of deaths in each stratum

† P values were calculated using the log-rank test in the univariate analysis.

‡ P values were obtained from the Cox hazards model with adjustment for age, sex, smoking status, tumor histology, KPS score, tumor stage, application of chemotherapy and radiotherapy dose.

To determine if the influence of *VEGF *SNPs was substantially affected by tumor stage, we further made stratified analyses by separating the patients into two subgroups, stages IIIa and IIIb. We found that none of the three SNPs showed significant influence on OS, except for the CT genotype of *VEGF *-460 T > C SNP, which was marginally associated with increased OS in NSCLC patients of stage IIIb (adjusted HR = 0.56; 95% CI, 0.30-1.05, *P *= 0.071 and other data not shown).

### *VEGF* haplotypes and NSCLC survival

We further explored the haplotypes to evaluate the combined effect of the three polymorphisms on NSCLC survivals. There were five haplotypes with frequencies > 5% among all cases, and other less common haplotypes (frequencies < 5%) were combined into one group. The five most common haplotypes in the patients were -460C/-634G/+936C (C-G-C), T-C-C, T-G-C, T-C-T, and C-C-C with the respective frequencies of 38.8%, 23.8%, 17.7%, 8.8% and 5.4%, which were similar to those reported in the other Caucasian populations [15]. However, we did not find a significant impact on OS from the other haplotypes, compared to the most common C-G-C haplotype (data not shown).

## Discussion

Several studies have reported the association between *VEGF *polymorphisms and progress and survival of different cancers [16-19], but no study has investigated the association between the *VEGF *polymorphisms and LA-NSCLC patients' survival to date. To reduce confounding effects of clinical parameters on the association, we limited our study subjects to a group of 124 Caucasian patients with homogenous stage IIIa and IIIb NSCLC, who received well-documented definitive chemoradiotherapy as previously described [13]. We found that the *VEGF *- 460 C variant genotypes were associated with a significantly improved OS, compared with the *VEGF *-460 TT genotype.

To give a comprehensive view of the association between *VEGF *genotypes and the prognosis of different cancer types, we summarized the published data in Table 3. Most of these studies confirmed an influence of *VEGF *SNPs on the overall survival of cancer patients. However, these studies differed substantially in their conclusions. Even for the same *VEGF *SNP, different risk allele was reported. Different ethnic populations might be one rational reason for the inconsistent results. Another possible explanation may be that the effects of VEGF are tumor-specific. The interaction of different therapeutic strategies with *VEGF *genotypes may also contribute to the diverse clinical outcomes. In NSCLC, the three studies (including ours) were not consistent in the *VEGF *risk alleles, suggesting that a further investigation was warranted. For example, Masago *et al*. reported an association between -460 C allele and a poorer survival of advanced NSCLC in Japanese patients [20], which was opposite to our findings in Caucasian patients. Numerous factors could have played a role in the ethnic discrepancy, including gene-gene interaction from different genetic background and gene-environmental interaction from different lifestyles. Even in the same ethnicity of Caucasians, the study by Heist *et al*. failed to find any significant association between -460 C allele and survival in 462 early-stage NSCLC patients, most of whom were surgically resected [9]. In that study, there were only 32 patients (7%) receiving radiation and three patients (0.6%) treated with chemotherapy. In contrast, the 124 LA-NSCLC patients of current study all received radiotherapy, and carriers of the C allele of -460 T > C polymorphism were found to benefit from radiotherapy. These findings, once validated in larger studies, will guide tailored therapeutics for individual patients.

**Table 3:** Summary of the influence of *VEGF *SNPs on cancer OS

First author	Year	Cancer	Country	Ethnicity	SNPs	No.	Risk allele
Guan (Current)	2010	LA-NSCLC	USA	Caucasian	-460T > C, -634G > C, and 936C > T	124	T for -460T > C
Formento [23]	2009	Head&neck	France	Caucasian	-460T > C, -634G > C, and 936C > T	49	None
Masago [20]	2009	Advanced NSCLC	Japan	Asian	-460T > C, -1154G > A, -2578C > A, 405G > C, and 936C > T	126	C for -460T > C, A for -1154G > A, and A for -2578C > A
Dassoulas [24]	2009	Colorectum	Greece	Caucasian	-460T > C, -634G > C, -1154G > A, -2578C > A, and 936C > T	312	T for -460T > C, G for -634G > C, C for -2578C > A, and C for 936C > T
Bradbury [25]	2009	Esophagus	Canada	Caucasian	-460T > C, 405G > C, and 936C > T	361	C for 936C > T
Heist [9]	2008	Early NSCLC	USA	Caucasian	-460T > C, 405G > C, and 936C > T	462	G for 405G > C and C for 936C > T
Kim [16]	2008	Colorectum	Korea	Asian	-634G > C, -2578C > A, and 936C > T	445	G for -634G > C and T for 936C > T
Kim [26]	2007	Stomach	Korea	Asian	-116G > A, -460T > C, 405G > C, and 936C > T	503	C for -460T > C and T for 936C > T
Kawai [27]	2007	Renal cell	Japan	Asian	-634G > C, -2578C > A, and -1154G > A	213	C for -2578C > A
Hefler [17]	2007	Ovarian	Austria	Caucasian	-634G > C, -1154G > A, and -2578C > A	563	None
Tzanakis [28]	2006	Stomach	Greece	Caucasian	-634G > C, -2578C > A, -1154G > A, and 936C > T	100	C for -634G > C
Lu [19]	2005	Breast	China	Asian	-460T > C, 405G > C, and 936C > T	1119	C for -460T > C, and G for 405G > C

It is not clear how the *VEGF *-460 C allele contributes to a better survival in LA-NSCLC patients. A previous *in vitro *study indicated that the T allele of the *VEGF *-460 T > C polymorphism located in the promoter of the *VEGF *gene was associated with a decreased *VEGF *promoter activity [10]. Hence, the *VEGF *-460 C allele may be associated with an increased VEGF expression, which would promote tumor angiogenesis. However, the majority of NSCLC patients included in the current study received chemotherapy in addition to radiotherapy (112 out of 124). It is possible that the increased tumor vasculature may enhance radiotherapy efficacy through inhibiting tumor radioresistance from radiation-induced hypoxia, or facilitate the delivery of chemotherapeutic agents to the tumor site and may have led to enhanced synergistic effect with radiotherapy. Or, the *VEGF *-460 C allele has some additional unknown biological functions, besides regulation of mRNA expression. Further mechanistic studies are required to clarify this issue.

The strength of this study is that we included patients with stage IIIa and IIIb only, who received radiotherapy mostly in the range of 60-70 Gy with detailed OS data. However, there are several limitations. First, the study could not address the mechanism of how the *VEGF *polymorphisms influence the survival outcomes of lung cancer patients. Previous study demonstrated a good correlation between TC/CC genotypes of the *VEGF *-460 T > C polymorphism and increased serum VEGF levels in colorectal cancer patients [21]. An increased serum VEGF expression was also observed in ovarian cancer patients carrying -634 C allele [22]. We are collecting related data to determine if there is such correlation between the *VEGF *polymorphisms and the VEGF protein levels in NSCLC patients. Secondly, we only included three common functional, promoter *VEGF *SNPs, which is far from comprehensive. Indeed, the *VEGF *gene is highly polymorphic with at least 140 variants reported to date http://www.ncbi.nlm.nih.gov/SNP/. Some important functional SNPs may be missed or the observed association may result from genetic linkages with other untyped SNPs. Thirdly, our sample size is not big enough to allow evaluation of interactions between the studied polymorphisms and dose of radiation therapy. For the same reason, there appeared a wide confidence interval in our stratified analyses by tumor stage and the significance was lost due to the reduced statistical power. Therefore, a complete investigation of tagging SNPs in larger samples may be necessary in future studies.

## Conclusion

In summary, we found that the *VEGF *-460 C allele may be associated with a better survival of LA-NSCLC patients treated with chemoradiotherapy. Future prospective studies with large sample sizes and better study designs are required to confirm our findings.

## Competing interests

The authors declare that they have no competing interests.

## Authors' contributions

XXG, QYW, and ZXL designed the study. XXG, ZSL and XLY performed the experiments. XXG, MY, and HZ analyzed the data. XXG and MY wrote the manuscript. LEW, MSO, and RK coordinated the data and helped to revise the manuscript. All authors read and approved the final manuscript.

## Pre-publication history

The pre-publication history for this paper can be accessed here:

http://www.biomedcentral.com/1471-2407/10/431/prepub
